# Assessing the Immunomodulatory Activity of Ethanol Extract of *Sambucus javanica* Berries and Leaves in Chloramphenicol-Induced Aplastic Anemia Mouse Model

**DOI:** 10.21315/tlsr2020.31.2.9

**Published:** 2020-08-06

**Authors:** Wira Eka Putra, Muhaimin Rifa’i

**Affiliations:** 1 Department of Biology, Faculty of Mathematics and Natural Sciences, Universitas Negeri Malang, Indonesia; 2 Department of Biotechnology, Faculty of Mathematics and Natural Sciences, Universitas Negeri Malang, Indonesia; 3 Department of Biology, Faculty of Mathematics and Natural Sciences, Brawijaya University, Indonesia

**Keywords:** Aplastic Anemia, Chloramphenicol, Immunomodulation, *S. javanica*

## Abstract

Aplastic anemia, life-threatened disease, is a hematologic disorder characterised by bone marrow hypoplasia. Multiple modalities such as bone marrow transplantation and immunosuppression treatment have been proposed to ameliorate this entity, however it remains ineffective. *Sambucus*, a group of herb plants, possesses a broad spectrum of medicinal properties such as antioxidant, insulin-like activity, anticancer and antiviral. However, the study about its activity toward aplastic anemia incidence is based on limited data. Thus, the research aim of this study was to evaluate the immunomodulatory activities of *Sambucus javanica* in chloramphenicol-induced anemia aplastic mouse model. In this present study, BALB/c mice were administrated with chloramphenicol (CMP) to induce aplastic anemia then followed by *S. javanica* extracts treatment. Additionally, cellular and molecular aspects were evaluated by flow cytometry and Hematoxylin-Eosin staining. Further analysis showed that *S. javanica* extracts could promote the population number of regulatory T-cells and naive cytotoxic T-cells. Moreover, those extract also reduced the inflammation and necrotic incidence in CMP-induced mouse aplastic anemia model. Together, these results suggest that *S. javanica* has therapeutically effect to aplastic anemia by altering the immune system as an immunomodulatory agent.

**Highlights**

- *S. javanica* extracts promoted CD4^+^CD25^+^CD62L^+^ regulatory T-cells and CD8^+^CD62L^+^ naive T-cells in aplastic anemia mice model.
- *S. javanica* extracts repressed inflammation rate of CD4^+^TNF-α^+^ cells and CD4^+^IFN-γ^+^ in aplastic anemia mice model.
- The immunomodulatory effect of *S. javanica* extracts reduces the incidence of necrosis in renal section of aplastic anemia mice model.

## INTRODUCTION

Aplastic anemia is a clinical syndrome which caused severe damage to hematopoietic precursor cell compartment in bone marrow (Muthukumaraswamy 2004). This entity leads to the other severe hematopoietic disorders such as anemia, neutropenia, and thrombocytopenia which considered as a harmful condition due to their effects causing the infection and bleeding (Rao 2014). Additionally, the International Aplastic Anemia and Agranulocytosis Study reported the incidence of acquired aplastic anemia is about two per a million persons per year (Segel & Lichtman 2010; Young & Kaufman 2008). Allogenic bone marrow transplantation or immunosuppression strategy is a standard way to treat aplastic anemia patients. However, most of the people are difficult to reach the access to this therapy due to the success rate and economic issue (Maluf *et al*. 2009).

*Sambucus* is a group of herbs that contains bioactive-rich compounds such as phenolic acid and flavonoid. Due to its compounds, *Sambucus* was considered as a medicinal plant for a long time ago by folk-society (Ulbricht *et al*. 2012; Kinoshita *et al*. 2012). Also, the previous investigation has revealed that *Sambucus* has been used for numerous medical treatments such as antioxidant, insulin-like activity, and antiviral referable to its role for inducing various immunomodulatory effects, including on cellular immunity, humoral immunity, hematopoiesis, and inflammation (Putra *et al.* 2019; Barsett *et al*. 2012). However, our knowledge of the role of *Sambucus* extracts especially in aplastic anemia mouse model is mostly based on insufficient data. Therefore, the objective of our study is to evaluate the immunomodulatory activities of *S. javanica* in CMP-induced anemia aplastic mouse model. Thus, we believe this study will serve as a base for future studies on a therapeutic strategy to ameliorate aplastic anemia.

## MATERIALS AND METHODS

### Materials Preparation

*Sambucus javanica* were purchased from Materia Medica Batu, The Ministry of Health Indonesia. Ethanol extraction was performed to collect crude compounds of *Sambucus javanica* berries and leaves. On the other hand, experimental BALB/c mice model were purchased from Laboratory of Animal, Gadjah Mada University under pathogen-free certification. All component of this study already assessed by Brawijaya University Ethics Committee with No. 330-KEP-UB.

### Experimental Treatment

In this study, there are eight groups of experimental treatment applied which consist of five mice per group. Three-month-old male mice were orally administrated with CMP (Sanbe Farma) to induce acquired-aplastic anemia. Moreover, berries and leaves extract of *S. javanica* were occupied for two weeks to treat mouse model. The experimental treatments in each group explained as follow: vehicle group; CMP group, 130 mg kg^−1^ BW; Berries Dose 1 group, CMP 130 mg kg^−1^ BW + berries extracts 50 mg kg^−1^ BW; Berries Dose 2 group, CMP 130 mg kg^−1^ BW + berries extracts 100 mg kg^−1^ BW; Berries Dose 3 group, CMP 130 mg kg^−1^ BW + berries extracts 200 mg kg^−1^ BW; Leaves Dose 1 group, CMP 130 mg kg^−1^ BW + leaves extracts 50 mg kg^−1^ BW; Leaves Dose 2 group, CMP 130 mg kg^−1^ BW + leaves extracts 100 mg kg^−1^ BW; Leaves Dose 3 group, CMP 130 mg kg^−1^ BW + leaves extracts 200 mg kg^−1^ BW.

### Immunostaining and Flow Cytometry

The spleen was isolated from experimental mice, then followed by homogenisation for intracellular and extracellular immunostaining procedure as similar as our previous study (Putra *et al*. 2015). Several antibodies were used such as anti-mouse CD4, anti-mouse CD8, anti-mouse CD62L, anti-mouse CD25, anti-mouse TNF-α, and anti-mouse IFN-γ (Biolegend). Flow cytometry analysis was accomplished by BD FACS CaliburTM (BD Bioscience).

### Hematoxylin and Eosin Staining

The paraffinised renal sections of the experimental animal were aimed to stain with Hematoxylin-Eosin (HE) staining with the similar protocol as our previous research (Putra *et al*. 2017). The HE staining steps were performed in this study such as deparaffinisation, hydration, hematoxylin staining, eosin counterstaining, dehydration, and clearing. The semi-quantitative measurement was conducted to evaluate necrotic incidence in renal cortex of mouse model.

### Statistical Analysis

In this study, statistical analysis was evaluated by one-way ANOVA followed by Tukey’s HSD test. Each data set was replicated and is shown as mean ± SD values. The *p*-value of < 0.05 was considered to be statistically significant between two different two sets of the data.

## RESULTS

### Immunomodulatory Activities of *S. javanica* in CMP-induced Anemia Aplastic Mouse Model

**Figure 1:**
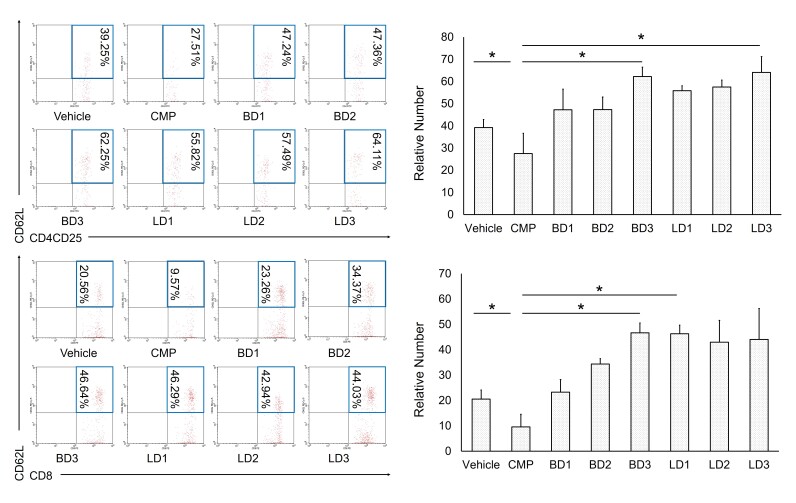
*S. javanica* extracts promoted CD4^+^CD25^+^CD62L^+^ regulatory T-cells and CD8^+^CD62L^+^ naive T-cells in aplastic anemia mice model. The representative flow cytometry diagrams show CD4^+^CD25^+^CD62L^+^ cells and CD8^+^CD62L^+^ cells expression. The bars are calculation of the relative number CD4^+^CD25^+^CD62L^+^ cells and CD8^+^CD62L^+^ cells expression. The results were represented as the mean ± SD. *p < 0.05, indicate significant difference. Treatment groups in this study were vehicle group; CMP group, 130 mg kg^−1^ BW; Berries Dose 1 group, CMP 130 mg kg^−1^ BW + berries extracts 50 mg kg^−1^ BW; Berries Dose 2 group, CMP 130 mg kg^−1^ BW + berries extracts 100 mg kg^−1^ BW; Berries Dose 3 group, CMP 130 mg kg^−1^ BW + berries extracts 200 mg kg^−1^ BW; Leaves Dose 1 group, CMP 130 mg kg^−1^ BW + leaves extracts 50 mg kg^−1^ BW; Leaves Dose 2 group, CMP 130 mg kg^−1^ BW + leaves extracts 100 mg kg^−1^ BW; Leaves Dose 3 group, CMP 130 mg kg^−1^ BW + leaves extracts 200 mg kg^−1^ BW.

The first set of analysis confirmed the impact of *S. javanica* extracts toward T-cell mediated disease population in CMP-induced anemia aplastic mouse model. Based on flow cytometry analysis, the relative number of CD4^+^CD25^+^CD62L^+^ regulatory T-cells were decreased after CMP treatment. However, the population number was significantly increased in berries and leaves extracts treatment. Moreover, there was a significant positive correlation between population number of regulatory T-cells naive cytotoxic T-cells. In this study, we demonstrated the population of CD8^+^CD62L^+^ naive cytotoxic T-cells was slightly decreased under CMP treatment, then in the similar pattern, it was up-regulated with berries and leaves extracts treatment of *S. javanica* (Fig. 1).

**Figure 2:**
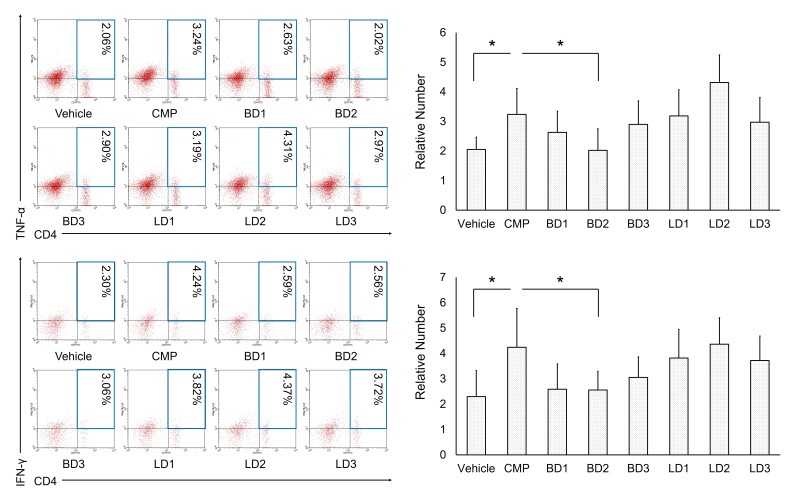
*S. javanica* extracts repressed inflammation in aplastic anemia mice model. The representative flow cytometry diagrams show CD4^+^TNF-α^+^ cells and CD4^+^IFN-γ^+^ cells expression. The bars are calculation of the relative number CD4^+^TNF-α^+^ cells and CD4^+^IFN-γ^+^ cells expression. The results were represented as the mean ± SD. *p < 0.05, indicate significant difference. Treatment groups in this study were vehicle group; CMP group, 130 mg kg^−1^ BW; Berries Dose 1 group, CMP 130 mg kg^−1^ BW + berries extracts 50 mg kg^−1^ BW; Berries Dose 2 group, CMP 130 mg kg^−1^ BW + berries extracts 100 mg kg^−1^ BW; Berries Dose 3 group, CMP 130 mg kg^−1^ BW + berries extracts 200 mg kg^−1^ BW; Leaves Dose 1 group, CMP 130 mg kg^−1^ BW + leaves extracts 50 mg kg^−1^ BW; Leaves Dose 2 group, CMP 130 mg kg^−1^ BW + leaves extracts 100 mg kg^−1^ BW; Leaves Dose 3 group, CMP 130 mg kg^−1^ BW + leaves extracts 200 mg kg^−1^ BW.

To assess inflammation incidence in an experimental mouse model, we evaluated the pro-inflammatory cytokines quantity such as TNF-α and IFN-γ that produced by CD4 T cells. As expected, our experiments depicted that the relative number of CD4^+^TNF-α^+^ or CD8^+^IFN-γ^+^ were dramatically up-regulated. On the other side, after berries extract treatment of *S. javanica* both of them are sharply down-regulated (Fig. 2). These results indicated that CMP induced inflammation and the *S. javanica* extracts might have an immunomodulatory effect.

### Necrosis in Renal of CMP-induced Anemia Aplastic Mouse Model

**Figure 3:**
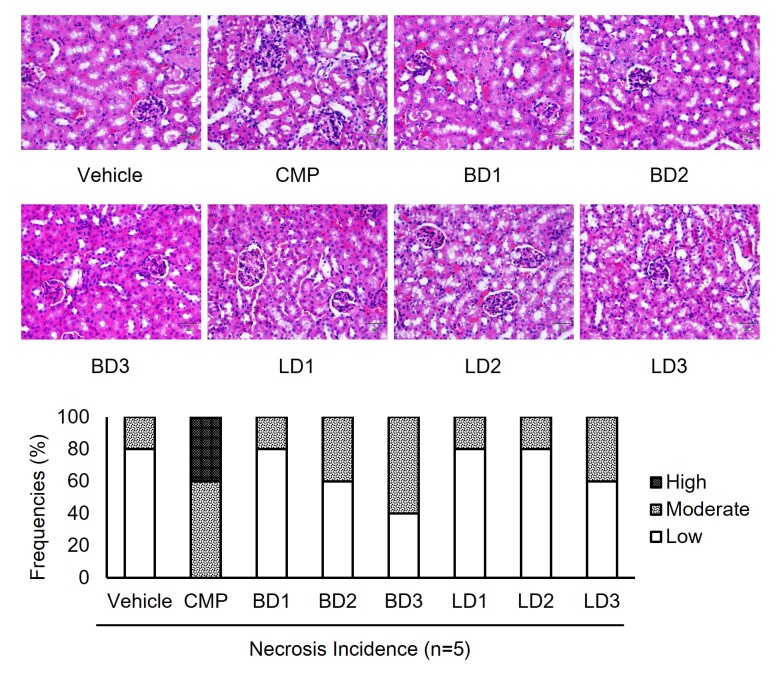
Representative microphotograph of renal section from aplastic anemia mice model (HE staining, M = 400×). Treatment groups in this study were vehicle group; CMP group, 130 mg kg^−1^ BW; Berries Dose 1 group, CMP 130 mg kg^−1^ BW + berries extracts 50 mg kg^−1^ BW; Berries Dose 2 group, CMP 130 mg kg^−1^ BW + berries extracts 100 mg kg^−1^ BW; Berries Dose 3 group, CMP 130 mg kg^−1^ BW + berries extracts 200 mg kg^−1^ BW; Leaves Dose 1 group, CMP 130 mg kg^−1^ BW + leaves extracts 50 mg kg^−1^ BW; Leaves Dose 2 group, CMP 130 mg kg^−1^ BW + leaves extracts 100 mg kg^−1^ BW; Leaves Dose 3 group, CMP 130 mg kg^−1^ BW + leaves extracts 200 mg kg^−1^ BW. Semi-quantitative calculation was performed to cluster the samples as high, moderate, or low of necrotic cells incidence. The bars are representative of necrotic cells incidence in renal samples of aplastic anemia mice model.

Semi-quantitative analyses between each experimental group were tested in this study. These tests highlighted that control positive underwent CMP treatment showing a high incidence of necrotic (Fig. 3). Together, this result revealed that drug-induced like CMP could promote necrosis cells in the renal cortex, however *S. javanica* extracts might exert their effect to ameliorate the cells.

## DISCUSSION

Aplastic anemia has been considered as an autoimmune disease confined by bone marrow destruction. This adverse effect naturally leads to hematopoietic progenitor cells disorder (Guinan 2011; Găman *et al*. 2009). Aplastic anemia is divided into two types, inherited and acquired aplastic anemia (Gupta *et al*. 2013). Specifically, acquired aplastic anemia is caused by chemicals or drugs induction, one of them is chloramphenicol (Rao 2014; Shukla *et al*. 2011; Yuan & Shi 2008). Hence, the patient’ symptoms of this disease are anemia, bleeding, and low level of reticulocytes (Găman *et al*. 2009).

*Sambucus* species were reported to have been considered for a long time ago as folk medicine. Almost all parts of *Sambucus* has been utilised such as berries, leaves, flowers and barks. It has been reported that *Sambucus* is bioactive-rich compounds such as flavonoids and polyphenols which possess a broad spectrum of biological properties including antioxidant, anti-viral, and anti-cancer (Gorchakova *et al*. 2007; Barak *et al*. 2002; Atkinson & Atkinson 2002). Similarly, in this study, we proved that berries or leaves of *S. javanica* crude extracts showed immunomodulatory activities by increased the population of T-cells mediated diseases such as regulatory T-cells and naive cytotoxic T-cells (Fig. 1). Immunomodulation is a complex biological mechanism that alters the immune system (Yeap *et al*. 2011). Generally, immunomodulatory actions are divided into two categories namely, immunostimulators and immunosuppressants (Abood 2017). Thus, immunomodulatory activities have a pivotal role to up or down regulate the immune system to fight and eliminate foreign substances that cause diseases, including hematological disorder (Nagarathna *et al*. 2013).

On the other hand, inflammation status which was evaluated by the decreasing number of pro-inflammatory cytokines production by helper T-cells (Fig. 2). Likewise, another study suggests that polyphenols and flavonoids can stimulate TNF-α and IFN-γ secretion which limiting inflammatory status. TNF-α plays a significant role in inflammation by which induce macrophage to produce other pro-inflammatory cytokines such as IL-1, IL-6 and IL12 (Badescu *et al*. 2015). Another pro-inflammatory cytokines, IFN-γ, has a complex immunomodulatory activity such as increasing antigens expression including MHC, viral and tumour antigens. Further, it also activates and improves the activities of macrophage, NK cells, and T-lymphocytes (Groza *et al*. 2010; Chen & Liu 2009). However, a study conducted by Badescu *et al*. (2015) found that *S. nigra* has immunomodulatory which exert their effect as anti-inflammation in a diabetic model.

**Figure 4:**
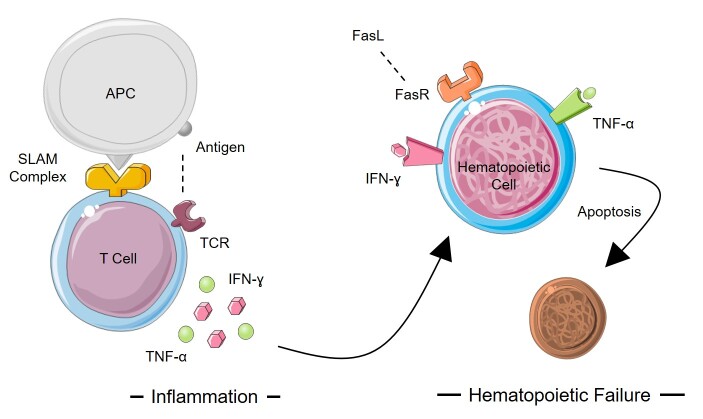
Hematopoietic progenitor cell failure’s indirect mechanism in acquired aplastic anemia. Antigen that presented by antigen presenting cell (APC) dramatically triggers T-cell activation followed by high production of pro-inflammatory cytokines. Moreover, IFN-γ and TNF-α induce activation of T-cell receptor (TCR) and FAS receptor (FASR). Furthermore, FASR activation can be elevated by FAS ligand (FASL) which leads to apoptotic incidence. On the other hand the effect of IFN-γ promoting NOS and producing NO which exert its toxic effect into the cells. Together, these steps significantly reducing cell cycling and causing cell death in hematopoietic progenitor cell.

Numerous investigations already reported that hematopoietic failures could be caused by inflammation. Thus, high secreted pro-inflammatory cytokines such as TNF-α and IFN-γ directly increase nitric oxidase (NO) production through nitric oxide synthase (NOS) activation (Fig. 4). Additionally, a report has shown that NO is toxic to the cell and promote cell death (Durga *et al*. 2014; Segel & Lichtman 2010). Furthermore, according to Ho *et al*. (2017) extracts from *S. nigra* flowers were known to inhibit NO secretion in macrophage and dendritic cells. Regarding that report, it suggests that *S. javanica* might exert their effect as well as *S. nigra* to reduce NO production.

Moreover, the most remarkable point to emerge from the data is the CMP administration can induce necrosis in the renal. However, the immunomodulatory effect of *S. javanica* extracts reduces the incidence of necrosis in experimental mouse (Fig. 3). Necrosis is defined as a premature death cell which characterised by shrinking-cytoplasm and condensed-nucleus. Additionally, other reports showed that necrosis could be caused by genetic, infection, chemical compounds, and other environmental factors (De Oliveira *et al*. 2013; Kaplowitz 2004). The CMP which is known as antimicrobial also has the potency to induce necrosis in renal. As an excretory organ, renal plays a crucial role in filtering unnecessary fluids including the remaining substances of chemical compounds. Likewise, Brenner and Stevens (2018) showed that renal toxicity is caused by synthetic drugs, including some derivate of antibiotics. A report showed that necrosis occurred might be a consequence of substances over-reactivity and mitochondrial dysfunction (Kaplowitz 2004). However, another observation conducted by Du *et al*. (2017) has revealed that phenolic compounds isolated from *Dioscorea zingiberensis* can prevent pancreatic necrosis induced by sodium taurocholate. Interestingly, Olaleye *et al*. (2014) also discovered the protective effect of flavonoids extracted from *Parinari curatellifolia* toward hepatic necrosis in acetaminophen-induced rats.

## CONCLUSION

This study has explained that leaves and berries extract of *S. javanica* exert their immunomodulatory activities by increasing the population number of regulatory T-cells and naive cytotoxic T-cells. Additionally, those extracts slightly decreased the inflammation and necrotic incidence in CMP-induced mouse aplastic anemia model. Finally, these results suggest that *S. javanica* has therapeutically effect to aplastic anemia by altering the immune system as an immunomodulatory agent.
